# Nanomaterials as catalysts for the sensitive and selective determination of diclofenac

**DOI:** 10.5599/admet.2116

**Published:** 2023-11-16

**Authors:** Totka Dodevska, Ivan Shterev

**Affiliations:** Department of Organic Chemistry and Inorganic Chemistry, University of Food Technologies, Plovdiv, Bulgaria

**Keywords:** electrochemical detection, electroanalysis, pharmaceutical analysis, drug analysis, environmental analysis

## Abstract

**Background and purpose:**

Diclofenac (DCF) is a non-steroidal anti-inflammatory drug possessing analgesic and antipyretic properties. It is used for the treatment of rheumatoid arthritis pain, osteoarthritis, and acute muscle pain conditions and can be administrated orally, topically or intravenously. Because of its widespread use, hydrophilicity, stability and poor degradation (bioaccumulation in the food chain), DCF is an emerging chemical contaminant that can cause adverse effects in the ecosystems. Taking into account the consumption of DCF in pharmaceutical formulations and its negative impact on the environment, the development of new sensitive, selective, cheap, fast, and online capable analytical devices is needed for on-site applications.

**Experimental approach:**

This brief review attempts to cover the recent developments related to the use of nanomaterials as catalysts for electrochemical determination of DCF in pharmaceutical formulations, biological fluids and environmental samples.

**Key results:**

The article aims to prove how electrochemical sensors represent reliable alternatives to conventional methods for DCF analysis.

**Conclusion:**

The manuscript highlights the progress in the development of electrochemical sensors for DCF detection. We have analyzed numerous recent papers (mainly since 2019) on sensors developed for the quantitative determination of DCF, indicating the limit of detection, linear range, stability, reproducibility, and analytical applications. Current challenges related to the sensor design and future perspectives are outlined.

## Introduction

**Figure 1.**
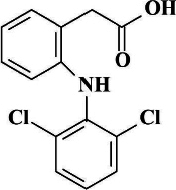
Chemical structure of DCF.

Diclofenac (DCF), 2-[2-(2,6-dichloroanilino) phenyl] acetic acid (Figure 1), is an anti-inflammatory drug with a strong antipyretic and analgesic effect. It is widely used in its sodium or potassium salt forms in clinical medicine for the treatment of inflammatory conditions such as rheumatoid arthritis, osteoarthritis, ankylosing spondylitis, joint pain, sports injuries, migraine, dysmenorrhea, urinary tract infections, chronic inflammation, *etc.* [1]. Diclofenac comes in several formulations and brands (Diclofenac, Voltaren, Diclac, Almiral, Cataflam, Cambia, Lofena, Zipsor), which are used for specific purposes and may contain different amounts of the drug. DCF is also frequently used in veterinary medicine (Diclocare-Vet, Surpass). DCF is administered topically, orally or by intramuscular injection; it is well tolerated and rarely produces gastrointestinal ulcerations or other side effects. Topical gel adsorption was found to be 6 to 7 %; the remaining part is either washed off the skin or attached to clothing. Of the orally administered dose, between 65 and 70 % is excreted in urine and 20–30 % in feces as the parent drug or metabolites [2].

In recent years, the production and consumption of DCF have increased to an estimated level of 940 tons per year worldwide [3]. The growth in the DCF market is attributed to the increasing prevalence of chronic diseases, the rising geriatric population, and the growing demand for pain relief drugs. Because of the extensive use of DCF in pharmaceutical formulations, its poor degradation, and incomplete elimination during wastewater treatment, DCF is found at various concentration levels in freshwater ecosystems worldwide [4]. Lonappan *et al.* summarized the status of DCF in the environment and reviewed the information about its consumption, occurrence, toxicity, resistance, persistence, and metabolites [5]. They have concluded that at environmentally relevant concentrations, such as nanograms per liter, DCF may cause chronic adverse effects on fish populations, damaging renal and gastrointestinal tissue. In fish (Japanese medaka, zebrafish), DCF adversely affected the growth in the egg phase and resulted in a significant reduction of hatchability and delay in hatching. DCF may accumulate in the liver, kidney, gills and muscle tissues of rainbow trout and can cause cytological alterations even at 1 μg L^−1^. DCF significantly induced lipid peroxidation (LPO) in mussels, indicating tissue damage. Its ecotoxicity is due to the continuous accumulation in the aquatic environment, and little is known about the long-term effects [4,5]. Thus, DCF is considered a novel class of aquatic chemical contaminant that can cause adverse effects on various organisms. DCF was included in the European Union Decision Watch List that requires its environmental monitoring in the member states. In 2019, DCF was classified as a high-priority substance to monitor in safeguarding the aquatic environment, indicating the adverse effects of DCF along the trophic chains, from aquatic organisms to mammals [3]. Moreover, DCF has been reported to be fatal for vultures and eagles. The collapse of vultures due to the consumption of carcasses containing residues of DCF affects the community structure of the ecosystem. Later studies reported that the ban on DCF for veterinary use in certain countries was an effective measure and the vulture population is on the rise.

The identified human metabolites of DCF (hydroxylated and methoxylated derivatives of DCF as well as glucuronide-conjugated forms) were found in urine and plasma. The major metabolites of DCF are hydroxy-metabolites (3′-hydroxydiclofenac, 4′-hydroxydiclofenac, 5′-hydroxydiclofenac). Studies on these metabolites' occurrence and toxicological effects in the environment are still poorly understood. Research suspected some metabolites could be potentially more toxic than the parent compound. Additionally, due to the presence of active groups in metabolites of DCF, there is a possibility of chemical interactions with other organics, inorganics, metals, *etc.* This process may lead to the formation of a "new emerging contaminant" of unknown properties.

Considering its importance to the pharmaceutical industry, as well as its negative impact on the organisms and environment, it is crucial to develop effective analytical methods to quantify DCF in different pharmaceutical formulations, biological samples, and aquatic environments at trace levels. The reliable quantitative assessment of DCF is essential not only for therapeutic purposes and drug production but also to assess its environmental risk and to verify and improve the wastewater treatment processes [4].

Numerous methods have been reported for DCF quantitative detection, mainly based on conventional analytical techniques, including spectrophotometry [6], high-performance liquid chromatography (HPLC) [7], and gas chromatography-mass spectrometry (GC-MS) [8]. Though some of these techniques offer high selectivity and accuracy, the associated high cost, time-consuming pretreatment steps, and need for skilled operators restrict their applications.

In contrast, electroanalytical techniques have many attractive features, such as high sensitivity, extremely low detection limit, wide linearity, quick response time, cheap instrumentation, favourable portability, and easy operation procedures [9,10,11]. Being robust and compatible with novel microfabrication technologies, electrochemical sensors offer the desired characteristics for point-of-care tests for monitoring various clinical, pharmaceutical and environmental analytes. This brief review highlights the progress in the development of electrochemical sensors for DCF detection. We have analyzed numerous recent papers (mainly since 2019) on sensors developed for the quantitative determination of DCF, indicating the limit of detection, linear range, stability, reproducibility, and analytical applications.

## Electrochemical sensing of diclofenac

**Figure 2.**
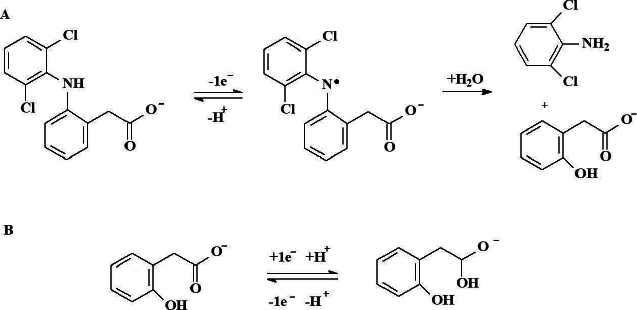
A) Oxidation mechanism for DCF anion. B) Mechanism for the electrochemical reduction of 2-(2hydroxyprop-2-enyl) acetic acid to 1-hydroxy-2-(hydroxyphenyl) ethanolate and its oxidation at pH 7.0.

Electrochemical sensors provide an affordable approach for fast, accurate, highly selective and sensitive quantitative determination of DCF. Aguilar-Lira *et al.* 2017 reported an investigation on the electrochemical behavior of DCF on a bare graphite electrode (GE) in phosphate buffer (PB, pH 7.0) [12]. By using cyclic voltammetry (CV), the authors confirmed that the electrochemical oxidation of DCF involves the formation of 2,6-dichloroaniline and 2-(2-hydroxyphenyl) acetic acid (Figure 2). An anodic peak was found at 0.557 V, reversing the cycle results in a new cathodic peak at 0.272 V, which is proposed to be related to the reduction of 2-(2hydroxyprop-2-enyl) acetic acid.

During the second cycle, a new cathodic peak is observed at 0.333 V, associated with the oxidation of 2-(2hydroxyprop-2-enyl) acetic acid to 1-hydroxy-2-(hydroxyphenyl) ethanоlate. Both electrochemical processes resulted in an adsorption-controlled, statistical fit of theoretical information with the experimental voltammograms. One-electron transfer is demonstrated, which supports the proposed mechanism. From an analytical point of view, it was demonstrated that direct quantification of DCF by CV using the anodic oxidation peak results less favorable when compared with an indirect quantification using the 2-(2hydroxyprop-2-enyl) acetic acid oxidation peak. With this knowledge, a new competitive DPV methodology for the indirect quantification of DCF in commercial drugs and urine samples was proposed. No significant differences were registered when comparing the results obtained with the proposed and official spectrophotometric methods. These results demonstrate that simple electrodes, such as graphite, can be useful for quantitative analysis of DCF when considering robust chemometrics.

Modification of the working surface has been extensively investigated to improve the electrode selectivity and sensitivity. A literature survey reveals that common working electrodes, such as metallic solid electrodes, glassy carbon electrodes (GCEs), carbon paste electrodes (CPEs), pencil graphite electrodes (PGEs), and screen-printed electrodes (SPEs), after appropriate modification, are effective at DCF sensing.

Biological and pharmaceutical samples contain many substances, which may affect the selectivity of the electrochemical sensor. Therefore, the research teams investigate as possible interferents ascorbic acid, uric acid, dopamine, various carbohydrates (glucose, fructose, lactose, sucrose), metal ions (Na^+^, K^+^, Ca^2+^, Mg^2+^, Fe^3+^), anions (Cl^−^, SO_4_^2−^), phenolic compounds with a similar structure as DCF (paracetamol, β-Estradiol), and L-histidine (supplement used to treat rheumatoid arthritis).

We have briefly reviewed the materials used to fabricate modified electrode surfaces to detect DCF. The fabrication techniques and analytical performance of various types of electrodes modified with metal nanoparticles (MNPs), metal oxide nanoparticles (MOxNPs) carbon nanomaterials such as carbon nanotubes (CNTs), graphene (GR), graphene oxide (GO), metal-organic frameworks (MOFs), polymers, *etc*. were summarized. The role of the nanomaterials in improving the electrochemical performance of the modified electrodes is discussed.

## Sensors based on modified glassy carbon electrodes

Glassy carbon electrode (GCE) is macroscopically isotropic with the same electrical properties in all directions, hard, chemically resistant, and of high density because the existing pores are tightly closed [13]. GCE is commonly used as a convenient platform in electrochemical applications because of its remarkable properties, such as excellent electrical conductivity, low background current, electrochemical inertness over a wide potential range, and ease of surface modification. However, employment of conventional GCE has been restricted because of its slow electron transfer kinetics and high overpotentials of the redox process. Thus, an increasing interest has been paid to the development of modified GCEs applicable to the electroanalysis of DCF [4,14-20].

Several papers reported on DCF electrochemical detection using carbon-based materials and/or metal nanoparticles as GCE modifiers [14-18]. Carbon nanotubes (CNTs) have become the subject of intense investigation since their discovery. CNTs appear to be promising active electrode materials due to their remarkable electrical, chemical, mechanical and structural properties. The unique properties of CNTs make them extremely attractive for electrochemical detection. They provide a sufficient number of active sites for the appropriate adsorption and subsequent development of the redox process involved in the detection of a target analyte. CNTs efficiently facilitate the electrochemical oxidation of DCF, establishing novel minimized and easily prepared sensing platforms for sensitive and selective detection.

Slim *et al.* proposed a COOH-functionalized multiwalled carbon nanotubes (MWCNTs) film-coated GCE toward DCF detection at neutral pH [14]. Carboxylated MWCNTs show good mechanical properties, high surface area, enhanced hydrophilicity, ability to promote electron transfer reactions and higher attachment properties due to the functional groups on their surface. Therefore, MWCNTs-COOH can help generate a more homogeneous surface with better electrochemical activity than MWCNTs. Upon modification of the GCE surface, the electrochemical peak current of DCF is enhanced owing to the increase in conductivity, porosity and surface area of the modified electrode. A linear calibration was achieved in a range of 2 to 15 μM and the limit of detection (LOD) was 0.1 μM DCF. The stability of the proposed sensor MWCNTs-COOH/GCE was examined for 10 days with acceptable RSD values.

**Figure 3.**
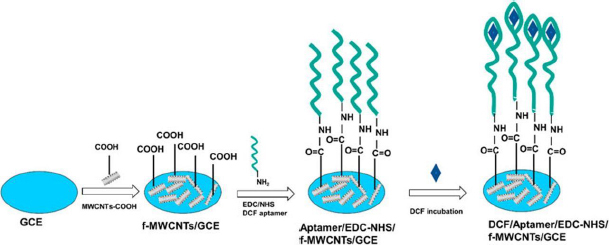
Schematic view of the DCF electrochemical aptasensor development. Reproduced from Ref. [4]. Permissions under Attribution 4.0 International (CC BY 4.0).

Functionalized MWCNTs also can be the base matrix for the immobilization of biomolecules to fabricate biosensors. Bioreceptors, such as aptamers (artificial single-stranded DNA molecules), give a particular affinity for the specific targets, providing extremely high selectivity of analysis [4]. In this work, authors have integrated the advantages of f-MWCNTs to be the matrix providing high grafting density of aptamers, specific biorecognition properties of the aptamers, and electrochemical methods for selective and sensitive quantification of DCF. The f-MWCNT nanocomposite and DCF aptamer were attached on the surface of GCE through the drop casting, and covalent amide bonds formed by the carboxylic acid groups on the f-MWCNTs and the amino groups from the DCF aptamer (Figure 3). The obtained aptasensor showed two linear concentration ranges for DCF detection, from 250 fM to 1 pM and from 1 pM to 500 nM, with an extremely low detection limit of 162 fM.

Nanometer-sized metal particles exhibit excellent electrocatalytic activity due to their relatively high surface area-to-volume ratio and interface-dominated properties, which significantly differ from their bulk counterparts. An important and attractive behavior of MNPs is the ability to tune and tailor their electrocatalytic activity by changing their size and shape. Afkhami *et al.* detected DCF using gold nanoparticles/multiwalled carbon nanotubes modified GCE (AuNPs/MWCNT/GCE), achieving LOD of 20 nM [15]. AuNPs with large surface area, stability, good biocompatibility, high conductivity and electrocatalytic characteristics have been used to improve the sensitivity and the limit of detection. According to the experimental results, the AuNPs/MWCNT hybrid can remarkably increase the electroactive surface area and enhance the electron transfer between the electrode and the analyte. The data showed that this modified electrode exhibits high catalytic activity towards the electrooxidation of DCF by significantly decreasing the oxidation overpotential and enhancing the peak currents. The proposed method has been applied to the voltammetric determination of DCF in real samples, such as commercial tablets and urine, with satisfactory results.

Reduced graphene oxide (rGO) is widely utilised, which is usually fabricated from the oxidation/exfoliation of graphite to graphene oxide (GO) and then its reduction to graphene via a variety of routes such as chemical, thermal or electrochemical [21]. Yu *et al.* 2021 described a simple strategy for the fabrication of a new modified electrode exploiting the synergetic effect of CuNPs and reduced graphene oxide (rGO) for the detection of DCF [16]. CV technique showed that CuNPs/rGO/GCE exhibited improved electrocatalytic activity and higher sensitivity for the determination of DCF than that of GCE, rGO/GCE and CuNPs/GCE. As an amperometric sensor, CuNPs/rGO/GCE shows extremely low detection limit (8 nM) and high electrode sensitivity in the concentration range from 20 to 400 μM. The applicability of CuNPs/rGO/GCE to determine DCF concentration in real pharmaceutical samples was examined and results showed that recovery (≥ 97.66 %) and RSD (≤ 3.05 %) values were acceptable, so this method provided suitable precision and accuracy for practical analyses.

ZnO core@Cu shell nanoparticles were synthesized by a wet chemical process and used to modify GCE by applying the drop-casting technique [17]. The electrooxidation of DCF was studied by CV and the concentration effect was read using square wave voltammetry (SWV) in the concentration range of about 0.01–300 μM. The sensor possesses qualities such as fast transfer of electrons, constancy, low detection limit (34 nM), high reproducibility, and stability. The pertinency of the proposed way was checked by quantification of DCF in pharmaceutical formulations and human urine samples. The authors reported that the mean of recovery of DCF in the tablet was 97.5 %, with an RSD of 0.7 %.

Recently, nanoporous materials of metal-organic frameworks (MOFs) consisting of inorganic metal clusters and organic molecules with coordination bonds have been used to develop electrochemical sensors. MOFs are innovative and emerging sensing platforms for the electrochemical determination of pharmaceuticals. They have attracted much attention due to their structural properties: a framework with a highly specific surface area, extremely high porosity, chemical stability, non-toxic nature, low density, and pore volume. Zeolite imidazolate framework-67 (ZIF-67) is a subclass of MOFs. It is an organic-inorganic hybrid solid with infinite and uniform crystalline coordination networks of cobalt ions (II) and imidazolate ligands [22]. Hoa *et al.* 2021 described the determination of DCF by using a GCE modified with ZIF-67 and graphitic carbon nitride (g-C_3_N_4_) [19]. The suspension of ZIF-67/g-C_3_N_4_ was cast dropwise on the GCE surface and dried. The fabricated ZIF-67/g-C_3_N_4_/GCE exhibits good electrocatalytic and accumulative effects on DCF. The modified electrode exhibits acceptable repeatability, reproducibility, and selectivity towards DCF. The proposed electrode allows determining DCF in human urine without pretreatment, and the results are comparable with those determined with HPLC. However, it must be emphasized that from a production viewpoint, the synthesis of MOFs, especially those comprising novel complex ligands, is time-consuming and expensive since it requires highly pristine organic precursors.

Drop casting methods are widely used to prepare the surface of chemically modified electrodes in which the modifying layer is composed of nanoparticles. Here, it should be pointed out that the drop-casting approach could be characterized by some drawbacks, such as low homogeneity and stability of the resulting modified surface. The evaporation of droplets containing suspended nanoparticles under ambient conditions forms a ring-like pattern. Тhis phenomenon has been labelled as the “coffee ring effect”, and its influence alters the distribution of the nanoparticles drop-casted on an electrode surface. Capillary forces, present as a result of solvent evaporation, push the modifier to the edges of the underlying electrode, thus, the periphery of the ring was seen to be concentrated with the solute particles in contrast to the centre of the stain. However, voltammetry as a sensing technique requires the formation of uniformly modified surfaces for which the coffee ring and its related effects present a significant limitation on the reproducibility of the drop-casted surfaces [23]. On the other hand, the agglomeration of particles upon electrode surface modification during the drop-casting method leads to a decrease in the fraction of electroactive nanoparticles. Additionally, the modifier physically adsorbed onto the electrode surface may be gradually stripped off in long-term operations. All these processes affect the sensing performance, reproducibility, and stability of the electrodes modified by drop-casting layers. Unfortunately, the authors [16,19] do not provide data on the long-term storage stability of the modified electrodes. The articles [16,17] do not contain data on the reproducibility of the proposed modification procedure.

Molecularly imprinted polymers (MIPs) serve as synthetic receptors for a wide variety of molecules. In general, the formation of MIP is based on the polymerization of one or more functional monomers with a cross-linker in the presence of a target molecule (template). After polymerization, the target molecule is extracted from the polymer matrix, which creates cavities in the structure with size, shape and interaction complementary to the template that can rebind reversibly and selectively in the presence of interferents [24]. Malekzadeh *et al.* 2020 developed a new MIP with nanoporous material of zirconium metal-organic frameworks (Zr-MOF/MIP/GCE) for selective and sensitive DCF analysis in a real sample (Alfen X tablet) [20].

Kokab *et al.*, for the first time, reported on the preparation of an electrochemical sensing platform that could selectively detect simultaneously three drugs (paracetamol PAR, DCF, and orphenadrine ORP) with ultrahigh sensitivity and LOD in the femtomolar range [25]. The platform comprises both acid- and base-functionalized CNTs with zinc oxide nanoparticles between the layers (COOH-CNTs/ZnO/NH_2_-CNTs). For authenticity of the accuracy of developed f-CNTs/ZnO/f-CNTs/GCE sensor in biological fluids, PAR, DCF, and ORP were concurrently analyzed in the three matrixes of each complex real sample (tablets, tap and drinking water, serum, urine, and artificial saliva/sweat).

**Figure 4.**
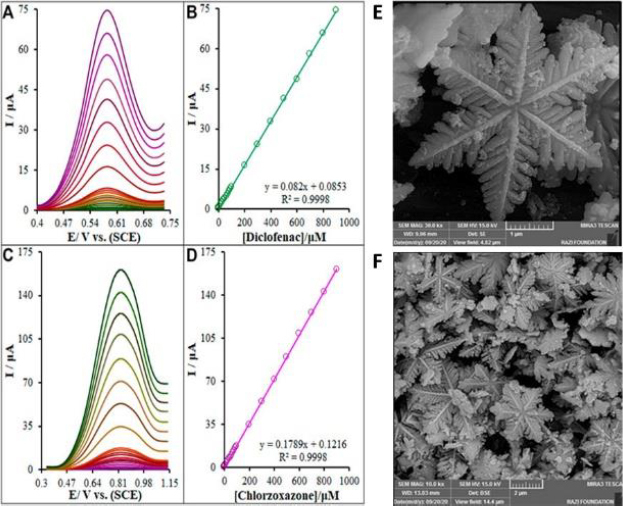
(A) and (C) DPVs of the La_2_O_3_@SF-L Cu_2_S/GCE in 0.1 M (pH 7.0) containing different concentrations of DCF and chlorzoxazone. (B) and (D) Corresponding calibration plots. (E) and (F) FESEM images of the La_2_O_3_ NP@SF-L Cu_2_S NS composite with 20 wt % La_2_O_3_. Reproduced from Ref. [26]. Permissions under Attribution 4.0 International (CC BY 4.0).

Next year, Baniahmad *et al*. reported the simultaneous detection of DCF and chlorzoxazone exploiting modified electrodes with novel composite – La_2_O_3_ NP@snowflake-like Cu_2_S nanostructure (Figure 4) [26].

The EDS analysis and the mapping images for this composite with 20 wt.% La_2_O_3_ confirmed uniform dispersion of each Cu, S, and La elements. The DPV findings showed a wide straight line (0.01–900 μM) with a LOD = 1.7 nM and a limit of quantification LOQ = 5.7 nM for the detection of DCF. The relative standard deviation of 0.94 % (*n*=6) confirmed the satisfactory repeatability of the proposed sensor. The intra-electrode and interelectrode RSD was 1.25 % and 2.83 %, respectively (n=6). The stability of the modified electrochemical sensor was analyzed as well. The electrodes were left in an ambient room for 3 weeks, and there was no significant fluctuation in peak current (2.6 %), confirming the appropriate stability of the La_2_O_3_@SF-L Cu_2_S/GCE. The electrochemical platform was used to monitor DCF and chlorzoxazone in drug tablets, human blood serum, and urine specimens.

Carbon paste electrodes (CPEs) have attracted great attention in electrochemistry due to their low-cost, non-toxic nature, simple preparation, renewable surface, and compatibility with various modifiers. CPEs can be modified to obtain novel electrochemical sensor platforms with desired, predefined properties [27,28]. Various techniques like electropolymerization, electrodeposition, drop-casting, and molecular imprinting were applied for the fabrication of electrochemical sensors. Electrochemical DCF sensors based on modified CPE as a transducer have been developed, which were able to detect low amounts of the target analyte [29-32].

Composite films with other materials, such as conducting polymers, ionic liquids, CNTs, MNPs, *etc*, are attractive materials for developing electrochemical sensors. A new sensor based on titanium oxide nanoparticles with ionic liquid [BMIM]Cl and coated by polymer (PEDOT) was developed for sensitive electrochemical determination of DCF [30]. The fabricated electrochemical platform based on PEDOT/TiO_2_/[BMIM]Cl/CPE, combined with the DPV technique, exhibits a linear current response in the concentration range from 5 to 100 μM, LOD of 11.7 nM, and LOQ of 39.1 μM, respectively. The good analytical performance of the sensor was confirmed for determining DCF in pharmaceutical formulation (Voltaren tablets), with good recoveries (97.98 to 102.05 %) and an acceptable relative standard deviation (RSD= 2.4 %).

Poly(L-methionine) modified carbon nanotube paste electrode (PMMCNTPE) was described as a platform for the enhanced sensitive determination of DCF [1]. The great improvement in the electroanalytical signal corresponding to the electrooxidation of DCF at PMMCNTPE clearly demonstrates that the polymeric layer of L-methionine acts as an adept promoter to boost the electrochemical kinetics of the reaction that takes place at the surface of the electrode. The compliance of the proposed sensor was validated by the assessment of the Voltaren tablet sample.

Motoc *et al.* [31] reported that simple integration of graphene within a multiwalled carbon nanotube paste electrode (GR-CNTPE) led to a stable and higher electrochemical response to DCF in 0.1 M Na_2_SO_4_ supporting electrolyte due to a larger electroactive surface area in comparison with multiwalled carbon nanotubes paste electrode (CNTPE). The authors found that the preconcentration step applied prior to DPV and multiple-pulsed amperometry (MPA) allowed for the enhancement of the electroanalytical performance of the DCF electrochemical detections, which were validated by testing in tap water. The lowest limit of detection of 1.40 ng L^−1^ was found using preconcentration prior to DPV under optimized operating conditions, which is better than that reached by other carbon-based electrodes reported in the literature. The selection of voltammetric or amperometric techniques for DCF detection with GR-CNTPE will consider the practical purpose concerning the water type and matrix. GR-CNTPE shows great utility for practical application in the development of the screening monitoring method for the determination of DCF in water samples (WWTP effluent, surface water, and drinking water).

Karimi-Maleh *et al.* [32] reported the first electrochemical sensor for simultaneous analysis of DCF, morphine and mefenamic acid. An electrochemical sensor was developed by modification of carbon paste electrodes using NiO-SWCNTs as conductive mediators and 2, 4-dimethyl-N/-[1-(2, 3-dihydroxy phenyl) methylidene] aniline (DDPM) as electrocatalyst. The NiO-SWCNTs/DDPM/CPE resolved the overlapping of DCF, morphine, and mefenamic acid signals and were used for selective analysis of these drugs in pharmaceutical and serum samples.

## Sensors based on modified screen-printed electrodes

The miniaturization of electrochemical systems is an issue that attracts increasing research attention because electronic measurements can be integrated into hand-held devices and smartphones [33,34]. The growing demand for easy-to-use analytical devices capable of rapidly providing valuable quantitative on-site information increases the interest in electrochemical sensors based on screen printed electrodes (SPEs) because of low cost, reduced size, portability, low reagent and sample consumption.

**Figure 5.**
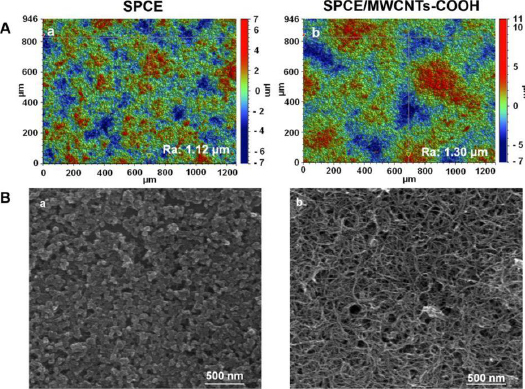
(A) Optical profiles. (B) SEM images of the SPCE (a) and the SPCE/MWCNTs-COOH (b). Reproduced from Ref. [35]. Licensee MDPI, Basel, Switzerland (2020).

A simple, sensitive and time-saving differential-pulse adsorptive stripping voltammetric (DPAdSV) procedure using a screen-printed carbon electrode modified with carboxyl functionalized MWCNTs (SPCE/MWCNTs-COOH) for the determination of DCF was presented by Sasal and co-workers [35]. The SPCE surface was covered with a thin layer of carboxyl-functionalized MWCNTs, which are also visible in the SEM images (Figure 5). The MWCNTs-COOH were dispersed onto the SPCE without aggregation with special three-dimensional structures and smooth surface. Under optimum conditions, very sensitive results were obtained with a linear range of 0.1 to 10 nM and a limit of detection value of 0.028 nM. According to the data presented, the SPCE/MWCNTs-COOH also exhibited satisfactory repeatability, reproducibility, and selectivity towards potential interferences. Moreover, for the first time, the electrochemical sensor was used for the determination of a real concentration of DCF (0.42 ± 0.08 nM) in environmental water samples without sample pretreatment steps.

Kimuam *et al.* introduced novel platinum nanoflowers and reduced graphene oxide nanocomposite-modified screen-printed electrodes (PtNFs/rGO/SPE) for sensitive and rapid detection of DCF in urine samples [36]. The nanocomposite of Pt and rGO was simultaneously synthesized via a single-step electrochemical reduction on the electrode surface. This method offers rapid and simple electrode fabrication with high preparation reproducibility. In the next year, a disposable electrochemical MIP sensor for the selective determination of DCF was constructed [37]. Its applicability was successfully demonstrated by the determination of DCF in spiked water samples (river and tap water). The proposed sensor and preparation methodology showed potential for mass production and to be customized for commercial applications.

Baezzat *et al.* fabricated MoS_2_ nanosheets modified graphite screen printed electrode (MoS_2_ NSS/GSPE) for the simultaneous detection of DCF and morphine [38]. In the same year, the electrochemical behavior of the DCF on GCE and SPCEs after an anodic activation (aGCE and aSPCEs) was studied [39]. This approach allowed to explore the analytical response of the two electrode types to DCF, highlight the differences between the two devices, and optimize the measurement conditions for both electrode types. Berto *et al.* concluded that the difference between the aGCE and the aSPCE lies in the interaction of the oxidation by-product with the electrode surface. Authors set the working procedure in order to obtain the best linear response: for the aGCE, they decided to oxidize DCF by an anodic potential (0.8 V for 30 s) and then measure the reduction peak of its by-product adsorbed on the electrode surface; on the other hand, for the aSPCEs, the determination is pursued by the direct analysis of the solution without a pre-oxidation/adsorption process. The approach reports lower detection limits than similar works, though the linear ranges are notably smaller. The researchers do not provide data on real sample analysis of DCF.

## Sensors based on other types of modified electrodes

The first paper-based platform for the determination of DCF was developed by Costa-Rama *et al*. [40]. The main novelty of this platform is that it integrated different steps, pre-concentration and detection, in a single device (lab-on-paper). This sensing platform was applied for DCF quantification in spiked tap water samples. Additionally, the versatility of this design enabled the fabrication of a multiplexed platform containing eight electrochemical cells that work independently. The low cost, small size and simplicity of the device allow on-site analysis, which is very useful for environmental monitoring.

An electrochemical sensor based on functionalized MWCNTs and gold–platinum bimetallic nanoparticles (AuPtNPs) modified gold electrode was developed for the determination of DCF in pharmaceutical and biological samples [41]. The authors reported a simple electrochemical procedure for the decoration of AuPt bimetallic nanoparticles on the carbon nanotubes, which are pre-casted on the surface of the gold electrode. Under the optimized experimental conditions, LOD was found to be 0.3 μM. However, this value is significantly higher than that of a number of other sensors. Also, the dynamic concentration range (0.5 to 1000 μM) is unsuitable for DCF analysis in water samples. The modified electrode AuPtNPs/MWCNTs/Au was successfully applied to determine DCF in tablet and urine samples.

The analytical parameters of different modified electrodes for the determination of DCF are compared in Table 1.

**Table 1.** Electrochemical sensors constructed on different sensing platforms for DCF detection in pharmaceutical, biological, and water samples.

Modified electrode	Method	Linear range (LOD)	Analytical application	Ref.	Year
MWCNTs-COOH/GCE	Amp.	2–15 μM (0.1 μM)	–	[14]	2019
AuNPs/MWCNT/GCE	SWV	0.03–200 μM (0.02 μM)	Tablet, urine	[15]	2016
Cu NPs/rGO/GCE	Amp.	20–400 μM (8 nM)	Tablets	[16]	2021
ZnO core@Cu shell/GCE	SWV	0.01–300 μM (34.1 nM)	Urine, tablets	[17]	2020
f-MWCNTs/NC/GCE	DPV	0.05–250 μM (0.02 μM)	Tablets, ampoule, urine, serum	[18]	2019
ZIF-67/g-C_3_N_4_/GCE	DPV	0.2–2.2 μM (71 nM)	Urine	[19]	2021
Zr-MOF/MIP/GCE	DPV	6.5–1500 μM (0.1 μM)	Tablets	[20]	2020
f-CNTs/ZnO/f-CNTs/GCE	SWASV	21–75 nM (78 fM)	Tablets, tap and drinking water, serum, urine, artificial saliva/sweat	[25]	2021
La_2_O_3_@SF-L Cu_2_S/GCE	DPV	0.01–900 μM (1.7 nM)	Tablets, serum, urine	[26]	2022
PEDOT/TiO_2_/[BMIM]Cl/CPE	DPV	55100 μM (11.7 nM)	Tablets	[30]	2018
GR-CNT CPE	DPV	1.01110 μg L^>1^ (1.4 ng L^>1^)	Tap water	[31]	2022
PtNFs/rGO/SPCE	DPV	0.1–100 μM (40 nM)	Urine	[36]	2020
MIP/SPCE	DPV	0.1010 μM (70 nM)	Tap and river water	[37]	2021
MoS_2_ NS_S_/GSPE	DPV	0.050600 μM (30 nM)	Tablets, urine	[38]	2022
a-GCE	DPV	0.01–0.05 μM (5.3 nM)	–	[39]	2022
a-SPCE		0.067–0.49 μM (24 nM)			
paper-based platform	LSV	0.1–100 μM (70 nM)	Tap water	[40]	2019
AuPtNPs/MWCNTs/Au	DPV	0.501000 μM (0.5 μM)	Tablets, urine	[41]	2019

## Advantages and limitations of electrochemical sensors for DCF quantification

A significant number of researchers have reported that the proposed electrochemical methodologies can be used with confidence to quantify DCF in samples like pharmaceutical ones and other more complex ones like urine and serum. When comparing the developed electrochemical method to the official spectrophotometric methodology, there are no significant differences between both methodologies, demonstrating that the electrochemical sensors are reliable for DCF quantification in real samples.

Electrochemical sensor devices have a number of advantages:

- Rapid analytical response (seconds);
- Quick data collection;
- High sensitivity and selectivity;
- Ultralow LODs and LOQs (in femtomalar range);
- Detection of the analyte is accomplished without previous separation;
- Ultra-low power consumption and extremely cost-effectiveness of the instrumentation (high benefit/cost ratio);
- Lightweight, easy to use and compact. Their analytical sensitivity is not compromised by miniaturization;
- Wireless network.

However, there are some shortcomings of electrochemical sensors:

- pH and ionic strength affect the sensor response (analysis should be performed in a specific buffer/electrolyte at an optimal pH value);
- Biofouling of active electrode surface – the result of the non-specific adsorption of proteins;
- Unsatisfactory long-term stability.

Current intensive research efforts are focused on resolving these challenges and unleashing the true potential of the electrochemical DCF sensors, as well as the commercialisation of cheap and long storage devices. The progress of electroanalytical systems for DCF detection requires a significant effort in fundamental, technical and medical studies. Solving these problems will require the combined expertise of electrochemists, material scientists, electronic engineers, pharmacists and biomedical scientists.

## Conclusion and outlook

This brief overview highlights the advances in the areas of electrode modification and successful strategies for signal amplification used in the electroanalytical systems for DCF monitoring. Data clearly show that the electrochemical sensors provide sensitive, rapid and affordable analytical platforms for the detection of DCF in pharmaceutical, biological, and water samples. The electrochemical methods combined with novel functional nanomaterials should provide a feasible path toward the next generation of portable, cost and energy-efficient sensing devices that would allow fast, accurate, and on-site detection of the target analyte.

After a thorough critical review of previous research studies, the following challenges were identified as priorities for the development of novel advanced electroanalytical devices for reliable quantitative detection of DCF.

✓ It is well known that the electrode fouling affects severely the analytical performance of sensor systems, such as their sensitivity, detection limit, reproducibility, and overall reliability. Therefore, the improvement of the selectivity of platforms for DCF detection with sensing applications in the field of medical science is imperative. Considering the high matrix complexity of blood serum and urine samples, innovative and efficient approaches should be developed to suppress the non-specific adsorption of interfering species. А possible approach is the surface functionalization of electrodes to enhance DCF detection and reduce biofouling, as well as the use of some electrochemical techniques to prevent this phenomenon, thus increasing the operational stability of the electrochemical sensors. Integrating such concepts into sensor design will pave the way to future point-of-care sensing systems due to more robust interfaces.
✓ Future developments in electrochemical design will inevitably focus on the technology of newly nano-sized materials to improve the sensitivity, reproducibility and stability of the analytical systems. Long-term reusability and maintaining the structural integrity of nanocomposites are significant challenges.
✓ More emphasis needs to be given to the theoretical analysis of catalyst synthesis. This will aid in establishing the correlation between the mechanism, structural morphology, catalytic activity and sensing ability of the electrocatalysts.
✓ Regarding the variety of ingredients considered for analytical control, multianalyte arrays will be much more useful in the field of pharmaceutical monitoring. Such electrochemical sensor devices should provide a fast, simple, simultaneous multianalyte assay.
✓ Producing commercially available sensors should extend their applications in extra-laboratory areas. The major challenge remaining for the commercial development of such applications is the requirement for a facile, reproducible, and low-cost production process for electrochemical devices on an economically beneficial scale while maintaining the accuracy achieved within a laboratory environment.
